# Sphingosine 1-phosphate (S1P) reduces hepatocyte growth factor-induced migration of hepatocellular carcinoma cells via S1P receptor 2

**DOI:** 10.1371/journal.pone.0209050

**Published:** 2018-12-13

**Authors:** Rie Matsushima-Nishiwaki, Noriko Yamada, Kouki Fukuchi, Osamu Kozawa

**Affiliations:** Department of Pharmacology, Gifu University Graduate School of Medicine, Gifu, Japan; Virginia Commonwealth University Medical Center, UNITED STATES

## Abstract

A bioactive lipid, sphingosine 1-phosphate (S1P), acts extracellularly as a potent mediator, and is implicated in the progression of various cancers including hepatocellular carcinoma (HCC). S1P exerts its functions by binding to five types of specific receptors, S1P receptor 1 (S1PR1), S1PR2, S1PR3, S1PR4 and S1PR5 on the plasma membrane. However, the exact roles of S1P and each S1PR in HCC cells remain to be clarified. In the present study, we investigated the effect of S1P on the hepatocyte growth factor (HGF)-induced migration of human HCC-derived HuH7 cells, and the involvement of each S1PR. S1P dose-dependently reduced the HGF-induced migration of HuH7 cells. We found that all S1PRs exist in the HuH7 cells. Among each selective agonist for five S1PRs, CYM5520, a selective S1PR2 agonist, significantly suppressed the HGF-induced HuH7 cell migration whereas selective agonists for S1PR1, S1PR3, S1PR4 or S1PR5 failed to affect the migration. The reduction of the HGF-induced migration by S1P was markedly reversed by treatment of JTE013, a selective antagonist for S1PR2, and S1PR2- siRNA. These results strongly suggest that S1P reduces the HGF-induced HCC cell migration via S1PR2. Our findings may provide a novel potential of S1PR2 to therapeutic strategy for metastasis of HCC.

## Introduction

Liver cancer is the second leading cause of cancer-related deaths [1]. Hepatocellular carcinoma (HCC) represents about 90% of primary liver cancer cases [1]. The survival rate of HCC is poor [1–3]. Due to improved diagnostic methods and prolonged survival of HCC patients, recurrence, intrahepatic or extrahepatic metastases of HCC are increased in the patients of HCC [2,3]. Even liver transplantation for the patients with early-stage HCC yields a 5-year survival rate of 70%, long-term survival is not prolonged [4]. The circulating HCC cells are the main cause of recurrence and metastasis after transplantation [5,6]. However, the details of the metastasis of HCC has not yet been clarified.

Sphingosine 1-phosphate (S1P) that is formed from sphingosine by sphingosine kinase, extracellularly plays as a potent bioactive lipid through its specific receptors on cell membranes, regulating various cell functions such as cell growth, survival, differentiation and migration [7–9]. S1P receptors (S1PRs) are classified as G protein-coupled receptors, known five S1PRs, S1PR1-5 [7–10]. It is recognized that S1PR1-3 are ubiquitously expressed in a variety of tissues, whereas S1PR4 is mainly expressed in the immune system, and S1PR5 is in the spleen and the central nervous system [9]. In liver, it has been shown that all five receptor subtypes, S1PR1-5 exist [11,12]. With regard to S1P and S1PRs in pathophysiology in the liver, it has been demonstrated that S1P is involved in hepatic insulin resistance via S1PR2, and enhances hepatic lipid storage via S1PR2 and S1PR3, and S1PR1 is responsible for the non-alcoholic fatty liver disease [9]. On the other hand, a high-fat diet rapidly develops fatty livers in the S1PR2 knock out mice than wild-type mice [13]. In addition, S1PR2 mediates glycogen synthase activation induced by conjugated bile acid [13]. The roles of S1P and S1PRs in HCC are still not precisely known.

Hepatocyte growth factor (HGF) is secreted by stellate cells and involved in hepatocyte regeneration after injury as a potent mitogen [14,15]. HGF is the unique ligand for c-mesenchymal epithelial transition factor receptor (c-MET), a tyrosine kinase receptor [14,15]. Accumulating evidence indicates that aberrant activation of tyrosine kinase receptors is related to the development of HCC [15]. Over-expression of c-MET and HGF is commonly shown in HCC, and elevated HGF levels predict poor prognosis [15]. In addition, the HGF/c-MET pathway plays a crucial role in HCC progression and metastasis [14]. As for the intracellular signaling system of HGF in HCC cells, it has been shown that HGF/c-MET signals are transduced through either mitogen-activated protein kinase (MAPK) or AKT signaling pathways, which involved in HCC cell migration and invasion [14,15]. In our previous study [16], we have demonstrated that HGF stimulates the proliferation of human HCC derived HuH7 cells via activation of the extracellular signal-regulated kinase (ERK), c-*Jun* N-terminal kinase (JNK) and AKT pathways [16]. However, details of HGF-induced migration of HCC cells remain unclear.

In this study, we investigated the effect of S1P on the HGF-induced migration of human HCC derived HuH7 cells, and the role of each S1PR. We herein show that S1P acts as a suppressive regulator in the HGF-induced HCC cell migration via S1PR2.

## Materials and methods

### Antibodies and chemicals

Recombinant human HGF was obtained from R&D Systems, Inc. (Minneapolis, MN). S1P was purchased from Sigma-Aldrich Co. (St. Louis, MO). SB203580 was purchased from EMD Millipore Co. (Billerica, MA). SP600125, PD98059 and Y27632 were obtained from Calbiochem-Novabiochem Co. (La Jolla, CA). Deguelin and glyceraldehyde-3-phosphate dehydrogenase (GAPDH) antibodies were purchased from Santa Cruz Biotechnology, Inc. (Santa Cruz, CA). SEW2871, CYM5520, CYM5541, CYM50260, A971432 and JTE013 were purchased from Tocris Bioscience (Bristol, UK). S1P, SB203580, SP600125, PD98059, deguelin, Y27632, SEW2871, CYM5520, CYM5541, CYM50260, A971432 and JTE013 were dissolved in dimethyl sulfoxide. Antibodies against phospho-p38 MAPK, phospho-myosin phosphatase targeting subunit 1 (MYPT-1), phospho-JNK, phospho-ERK and phospho-AKT (T308) were obtained from Cell Signaling Techenology, Inc. (Danvers, MA). Antibodies against S1PR1, S1PR2 and S1PR5 were obtained from Proteintech Group, Inc. (Rosemont, IL). Antibodies against S1PR3 and S1PR4 were purchased from Assay Biotechnology Company, Inc. (Fremont, CA) and Abgent, Inc. (San Diego, CA), respectively. An ECL Western blotting detection system was obtained from GE Healthcare UK Ltd. (Buckinghamshire, UK). Negative control-small interfering RNA (siRNA) (siGENOME Non-targeting siRNA Pool #2) and S1PR2-siRNA (siGENOME Human S1PR2 (9294) siRNA-SMART pool) were obtained from Dharmacon, a Horizon Discovery Group Co. (Cambridge, United Kingdom). Other materials and chemicals were obtained from commercial sources. The maximum concentration of dimethyl sulfoxide was 0.2%, which did not affect cell migration assay or Western blot analysis.

### Cell culture

Human HCC-derived HuH7 cells (JCRB0403) were obtained from the JCRB Cell Bank (Tokyo, Japan) [17]. The cells were maintained in Roswell Park Memorial Institute (RPMI) 1640 medium (Sigma-Aldrich Co.) containing 10% fetal calf serum (FCS; Hyclone Co., Logan, UT) at 37°C in a humidified atmosphere of 5% CO_2_/95% air. The cells were seeded into 100-mm diameter dishes (7 x 10^5^ cells/dish) in RPMI 1640 medium containing 10% FCS. After 3 days, the medium was exchanged for serum-free RPMI 1640 medium. After 24 h, the cells were used for Western blot analysis. For cell migration assay, the cultured cells were seeded into 100-mm diameter dishes (4 x 10^5^ cells/dish) in RPMI 1640 medium containing 10% FCS for 4 days, and then used for the experiments.

### Cell migration assay

A transwell cell migration assay was performed using Boyden chamber (polycarbonate membrane with 8-μm pores, Transwell, Corning Costar Co., Cambridge, MA) as described previously [18]. In brief, the cultured cells were seeded (1 x 10^5^ cells/well) onto the upper chamber in the serum-free RPMI-1640 medium. When indicated, the cells were pretreated with SB203580, SP600125, PD98059, deguelin, Y27632, S1P, SEW2871, CYM5520, CYM5541, CYM50260 or A971432 in the upper chamber for 60 min at 37°C. Then, HGF (30 ng/ml) was added to the lower chamber for 23 h at 37°C. In the case of JTE013, the cells were pretreated with JTE013 for 10 min in the upper chamber prior to S1P treatment. After the incubation with HGF, the cells on the upper surface of the membrane were mechanically removed. The migrated cells adherent to the underside of the membrane were fixed with 4% paraformaldehyde and stained with 4’,6-diamidino-2-phenylindole (DAPI) solution. The migrated cells were then photographed and counted using fluorescent microscopy at a magnification of 20× by counting the stained cells from three randomly chosen high power fields.

### Western blot analysis

The cultured cells were stimulated by 30 ng/ml of HGF or vehicle for the indicated periods. When indicated, the cells were pretreated with SB203580, SP600125, PD98059, deguelin or Y27632 for 60 min at 37°C. The cells were washed with phosphate-buffered saline, and then lysed and sonicated in a lysis buffer containing 62.5 mM Tris/HCl, pH 6.8, 2% sodium dodecyl sulfate (SDS), 50 mM dithiothreitol and 10% glycerol. SDS-polyacrylamide gel electrophoresis (PAGE) was performed by the method of Laemmli [19]. A Western blot analysis was performed as described previously [16,18,20] using phospho-specific p38 MAPK antibodies, phospho-specific MYPT-1 antibodies, phospho-specific JNK antibodies, phospho-specific ERK antibodies, phospho-specific AKT antibodies and GAPDH antibodies as primary antibodies with peroxidase-labeled anti-rabbit IgG antibodies (Cell Signaling Technology, Inc.) being used as secondary antibodies. The peroxidase activity on polyvinylidene difluoride membrane was visualized on X-ray film using the ECL Western blotting detection system.

### Cell counting assay

For cell counting, the HuH7 cells were plated on 96-well dish (3 x 10^3^ cells /well) in RPMI-1640 medium containing 10% FCS. Twenty-four hours after seeding, the medium of the cells was changed to RPMI-1640 medium without FCS, and treated with 10 μM S1P or vehicle for another 24 h. The cell numbers were then counted using a Cell Counting Kit-8 (Dojindo Laboratories, Kumamoto, Japan) according to the manufacturer’s instruction.

### siRNA transfection

For S1PR2 knockdown in HuH7 cells, the cells were transfected with S1PR2-siRNA (siGENOME Human S1PR2 (9294) siRNA-SMART pool) or negative control non-targeting siRNA using siLentFect Lipid Reagent (Bio Rad Laboratories, Inc., Hercules, CA, USA) in accordance with the manufacturer’s instruction. The cells were incubated with 50 nM siRNA-siLentFect complexes for 24 h. The transfected cells were subsequently harvested and used for the cell migration assay.

### Statistical analysis

The data are presented as the mean ± standard deviation (SD) of triplicate determinations from three independent cell preparations. The statistical significance of the data was analyzed using Student’s *t*-test. The values of *p*<0.05 were considered to be statistically significant.

## Results

### Effects of SB203580, SP600125, PD98059, deguelin or Y27632 on HGF-induced migration of HuH7 cells

In our recent study [18], we have shown that HGF stimulates the migration of HuH7 cells by a transwell cell migration assay. In this study, we found that HGF stimulated the cell migration dose-dependently over the range 1–70 ng/ml, and that the maximum effect of HGF was observed at 30 ng/ml (S1 Fig). Therefore, we used HGF at a concentration of 30 ng/ml to investigate the S1P-effect on the cell migration.

**Fig 1:**
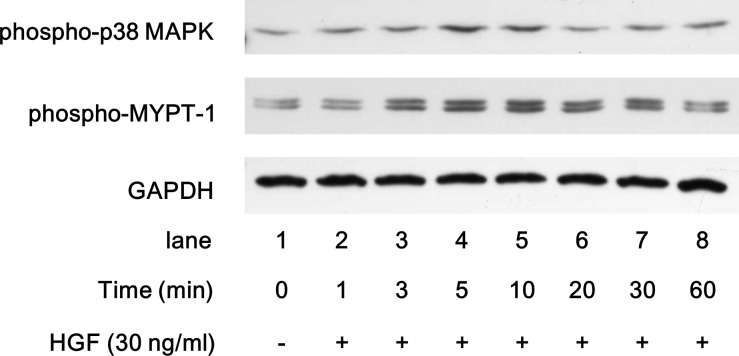
Effects of HGF on the phosphorylation of p38 MAPK and MYPT-1 in HuH7 cells. The cultured cells were stimulated with 30 ng/ml HGF for the indicated periods. The cell extracts were then subjected to SDS-PAGE with subsequent Western blot analysis using antibodies against phospho-specific p38 MAPK, phospho-specific MYPT-1 or GAPDH.

**Fig 2:**
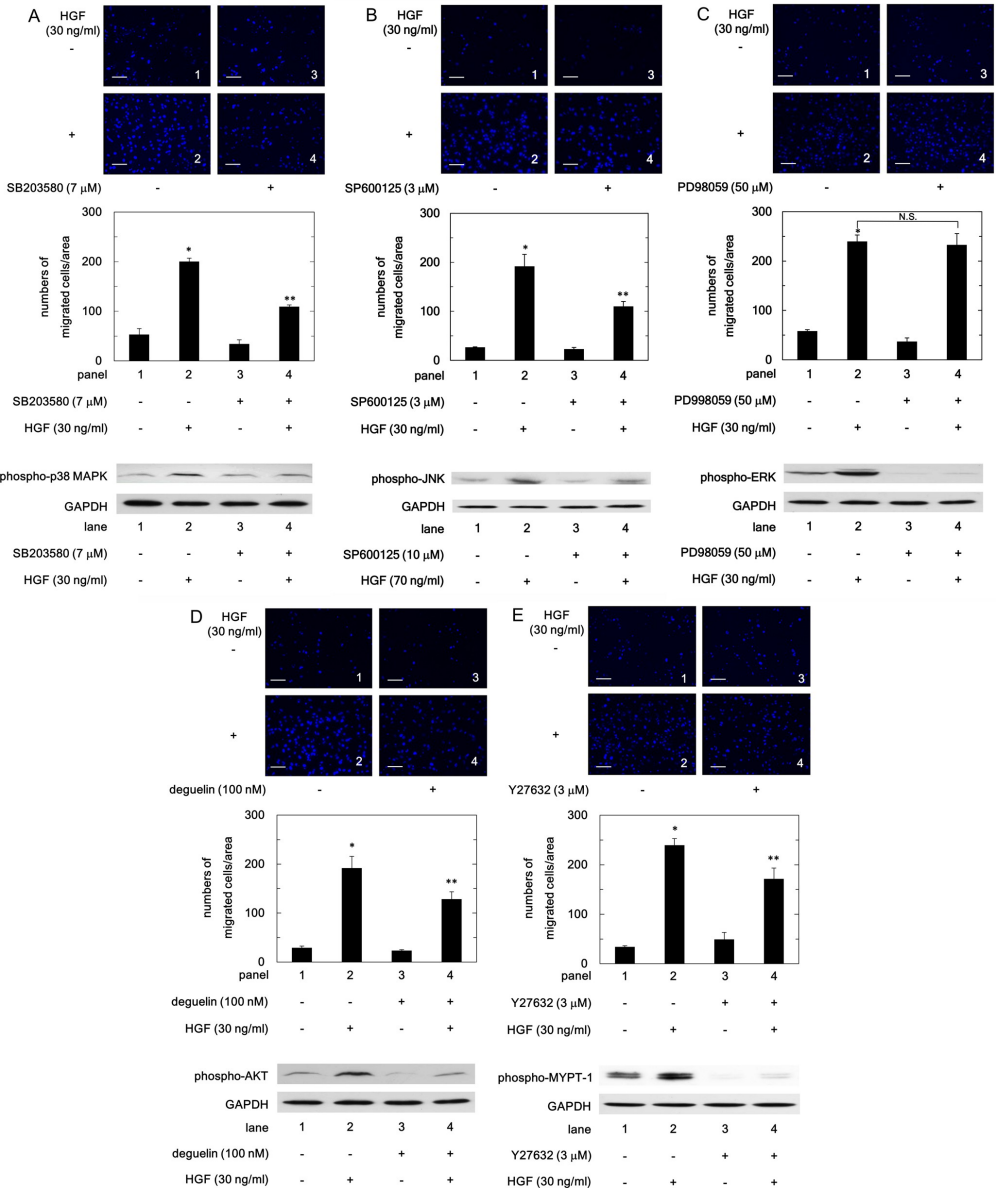
**Effects of SB203580, SP600125, PD98059, deguelin or Y27632 on the HGF-induced migration, and phosphorylation of p38 MAPK (A), JNK (B), ERK (C), AKT (D) and MYPT-1 (E) in HuH7 cells.** For the cell migration, the cells were pretreated with the indicated doses of SB203580 (A), SP600125 (B), PD98059 (C), deguelin (D) or Y27632 (E) for 60 min, and then stimulated by 30 ng/ml of HGF or vehicle for 23 h. The migrated cells were fixed with paraformaldehyde, and stained with DAPI for nucleus (blue signal). The cells were photographed by fluorescent microscopy at a magnification of 20× (upper panel) and counted (bar graph). For the Western blot analysis, the cells were pretreated with the indicated doses of SB203580 (A), SP600125 (B), PD98059 (C), deguelin (D) or Y27632 (E) for 60 min, and then stimulated by 30 ng/ml of HGF or vehicle for 5 min ((A), (B) and (E)) or 3 min ((C) and (D)). Each value represents the mean ± SD of triplicate determinations from three independent cell preparations. * *p*<0.05 compared to the value of the control cells without HGF. **p<0.05 compared to the value of the cells with HGF alone. N.S. designates no significant difference between the indicated pairs. Scale bar: 100 μm.

With regard to the intracellular signaling system of HGF, we have previously reported that HGF induces the activation of JNK, ERK and AKT in human HCC-derived HuH7 cells [16]. It is generally recognized that HGF activates several pathways including MAPK and AKT pathways, leading to the regulation of HCC survival, proliferation and migration [15]. In addition to MAPK and AKT pathways, it has been shown that Rho-kinase is implicated in HCC cell functions [21]. In this study, we found that HGF induced the phosphorylation of p38 MAPK and MYPT-1, a down-stream substrate of Rho-kinase in HuH7 cells, suggesting that p38 MAPK and Rho-kinase are activated by HGF (Fig 1). Therefore, we investigated the involvement of p38 MAPK, JNK, ERK, AKT or Rho-kinase in the HGF-induced HuH7 cell migration. SB203580, a p38 MAPK inhibitor [22], and SP600125, a JNK inhibitor [23], significantly attenuated the HGF-induced migration of HuH7 cells (Fig 2A and 2B, respectively, upper panels and bar graphs). However, PD98059, an inhibitor of the upstream kinase activating ERK [24], failed to inhibit the HGF-induced migration of HuH7 cells (Fig 2C, upper panels and bar graph). In addition, the HGF-induced cell migration was significantly suppressed by deguelin, an AKT inhibitor [25], and Y27632, a Rho-kinase inhibitor [26] (Fig 2D and 2E, respectively, upper panels and bar graphs). Furthermore, we confirmed that SB203580, SP600125, PD98059, deguelin and Y27632 truly inhibit the HGF-induced phosphorylation of p38 MAPK, JNK, ERK, AKT and MYPT-1 in HuH7 cells, respectively (Fig 2A–2E, each lower panel).

### Effect of S1P on the HGF-induced migration of HuH7 cells

**Fig 3:**
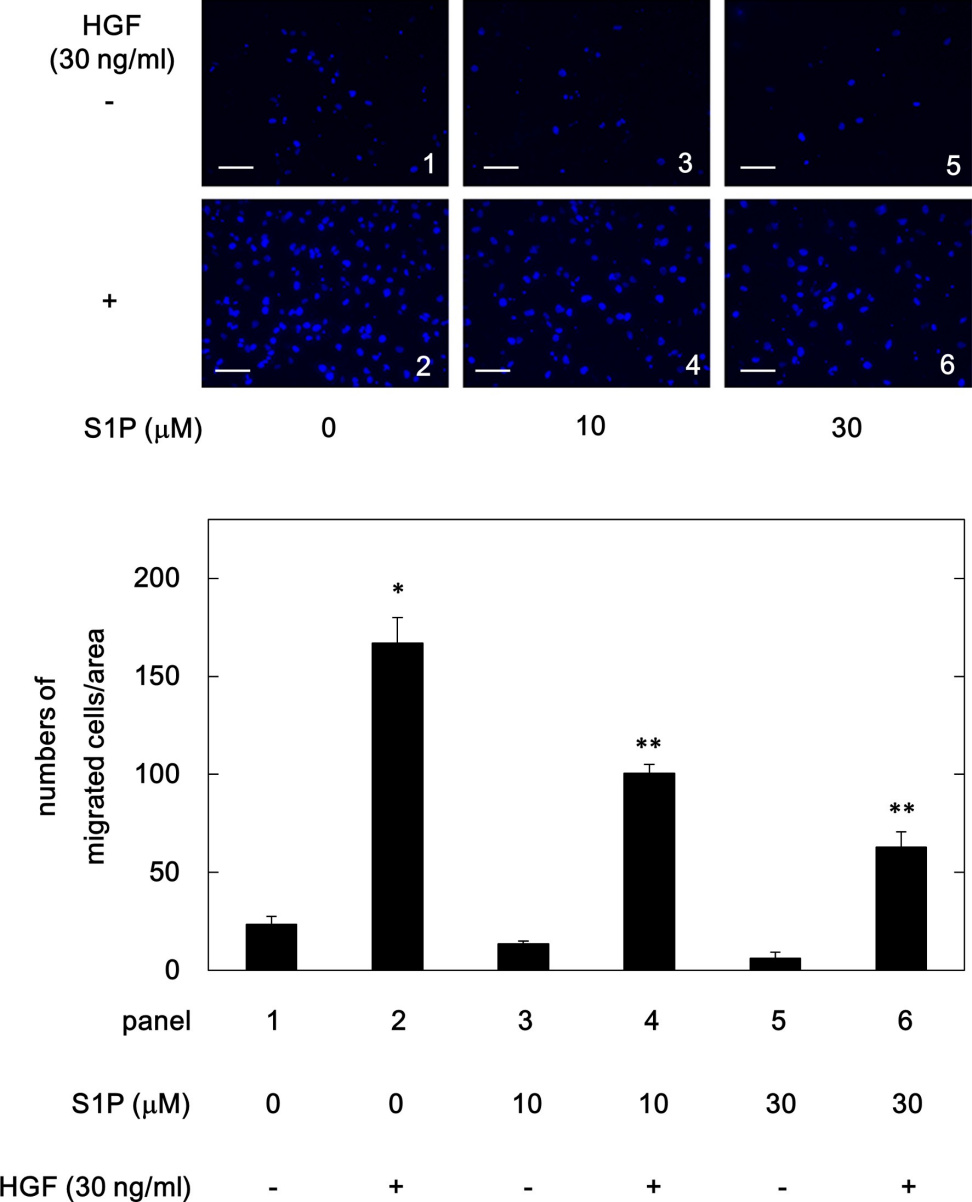
Effect of S1P on the HGF-induced migration of HuH7 cells. The cells were pretreated with the indicated doses of S1P for 60 min, and then stimulated by 30 ng/ml of HGF or vehicle for 23 h. The migrated cells were fixed with paraformaldehyde, and stained with DAPI for nucleus (blue signal). The cells were photographed by fluorescent microscopy at a magnification of 20× (upper panel) and counted (bar graph). Each value represents the mean ± SD of triplicate determinations from three independent cell preparations. *p<0.05 compared to the value of the control cells without HGF. **p<0.05 compared to the value of the cells with HGF alone. Scale bar: 100 μm.

We next examined whether S1P affects the HGF-induced HuH7 cell migration. As shown in Fig 3, S1P, which alone did not affect the cell migration, significantly suppressed the HGF (30 ng/ml)-induced migration of HuH7 cells in a dose-dependent manner over the range 10 μM and 30 μM. We confirmed that the cell viability of S1P (10 μM) treated HuH7 cells for 24 h was not significantly different from the control cells (Table 1). S1P (30 μM) caused approximately 60% reduction in the HGF-effect on HuH7 cell migration.

**Table 1:** The cell viability of S1P-treated HuH7 cells.

	Control	S1P (10 μM)
Cell viability (%)	100 ± 10.7	91.2 ± 8.4^a^

The data are the mean ± SD (n = 6).

^a^no significant difference compared to the control.

### Effects of agonists of S1PR1-5 on the HGF-induced migration of HuH7 cells

**Fig 4:**
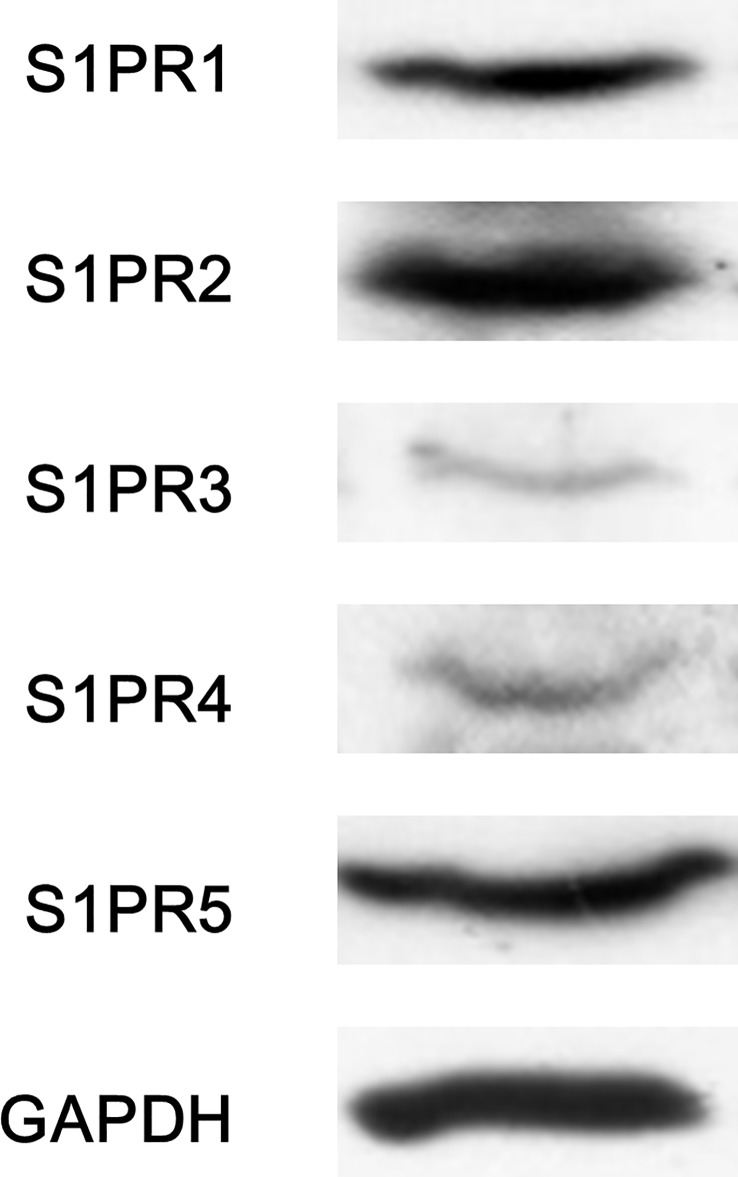
S1PRs in HuH7 cells. The HuH7 cell extract was subjected to SDS-PAGE with subsequent Western blot analysis using antibodies against S1PR1, S1PR2, S1PR3, S1PR4, S1PR5 or GAPDH.

**Fig 5:**
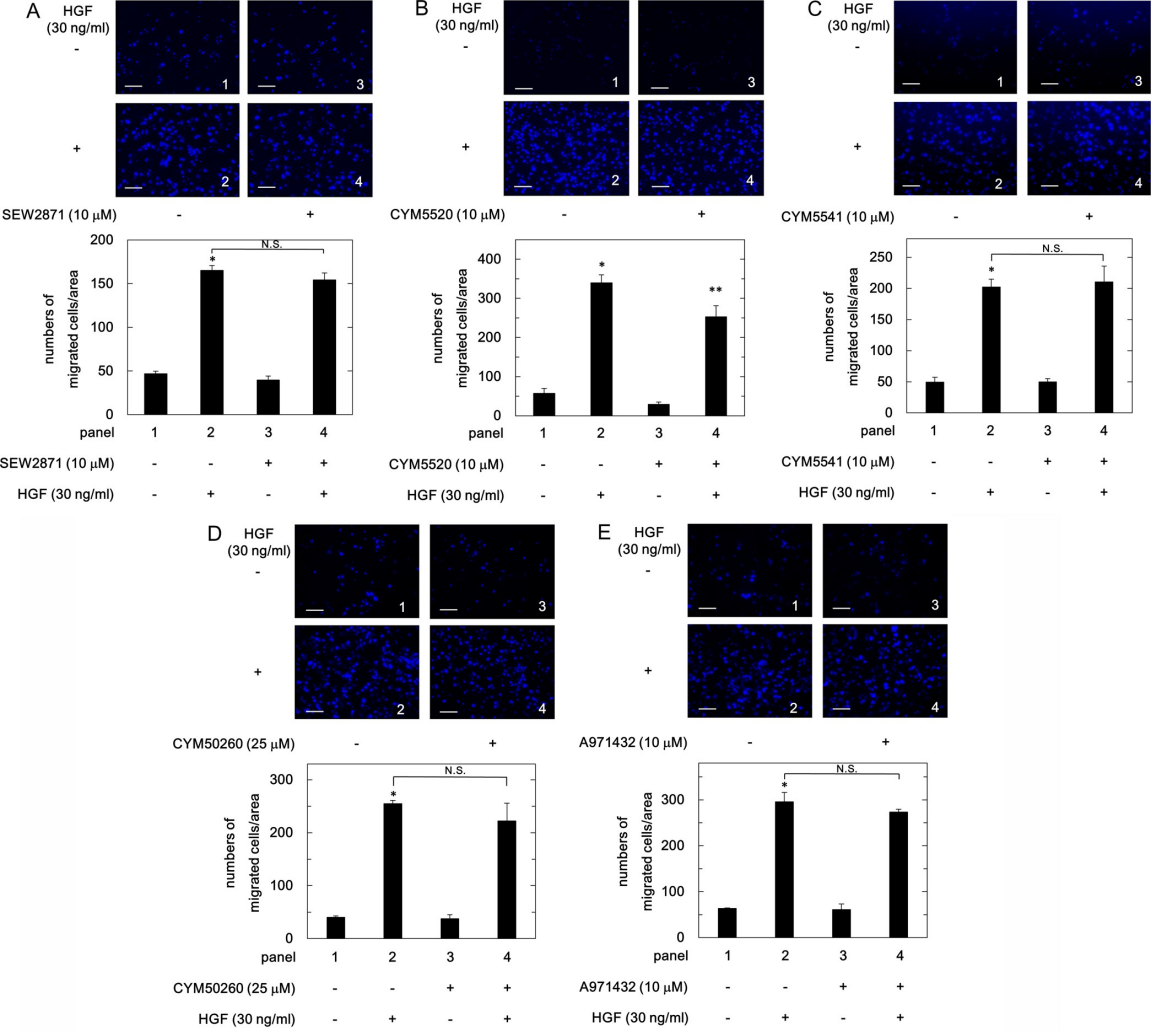
Effect of agonists of S1PR1-5 on the HGF-induced migration of HuH7 cells. The cells were pretreated with the indicated doses of SEW2871 (A), CYM5520 (B), CYM5541 (C), CYM50260 (D) or A971432 (E) for 60 min, and then stimulated by 30 ng/ml of HGF or vehicle for 23 h. The migrated cells were fixed with paraformaldehyde, and stained with DAPI for nucleus (blue signal). The cells were photographed by fluorescent microscopy at a magnification of 20× (upper panel) and counted (bar panel). Each value represents the mean ± SD of triplicate determinations from three independent cell preparations. *p<0.05 compared to the value of the control cells without HGF. **p<0.05 compared to the value of the cells with HGF alone. N.S. designates no significant difference between the indicated pairs. Scale bar: 100 μm.

The extracellular effects of S1P are exerted through specific receptors, five known receptors (S1PR1-5) [7–9]. It has been reported that all five receptors are expressed in human liver tissue [12]. We found that human HCC-derived HuH7 cells truly express S1PR2–5 (Fig 4). In order to investigate which S1PR mediates the suppressive effect of S1P on the HGF-induced HuH7 cell migration, we examined the effect of a specific agonist of each S1PR on the cell migration. However, SEW2871 (a selective agonist of S1PR1) [27], CYM5541 (a selective agonist of S1PR3) [28], CYM50260 (a selective agonist of S1PR4) [29] and A971432 (a selective agonist of S1PR5) [30] failed to reduce the HGF (30 ng/ml)-stimulated HuH7 cell migration (Fig 5A, 5C, 5D and 5E, respectively). On the contrary, CYM5520, a selective agonist of S1PR2 [31], significantly suppressed the HGF-induced migration of HuH7 cells (Fig 5B), suggesting that among five specific receptors, only S1PR2 mediates the S1P-signal on the HGF-induced migration of HuH7 cells.

### Effect of JTE013, a selective antagonist of S1PR2 on the suppression by S1P of HGF-induced migration of HuH7 cells

**Fig 6:**
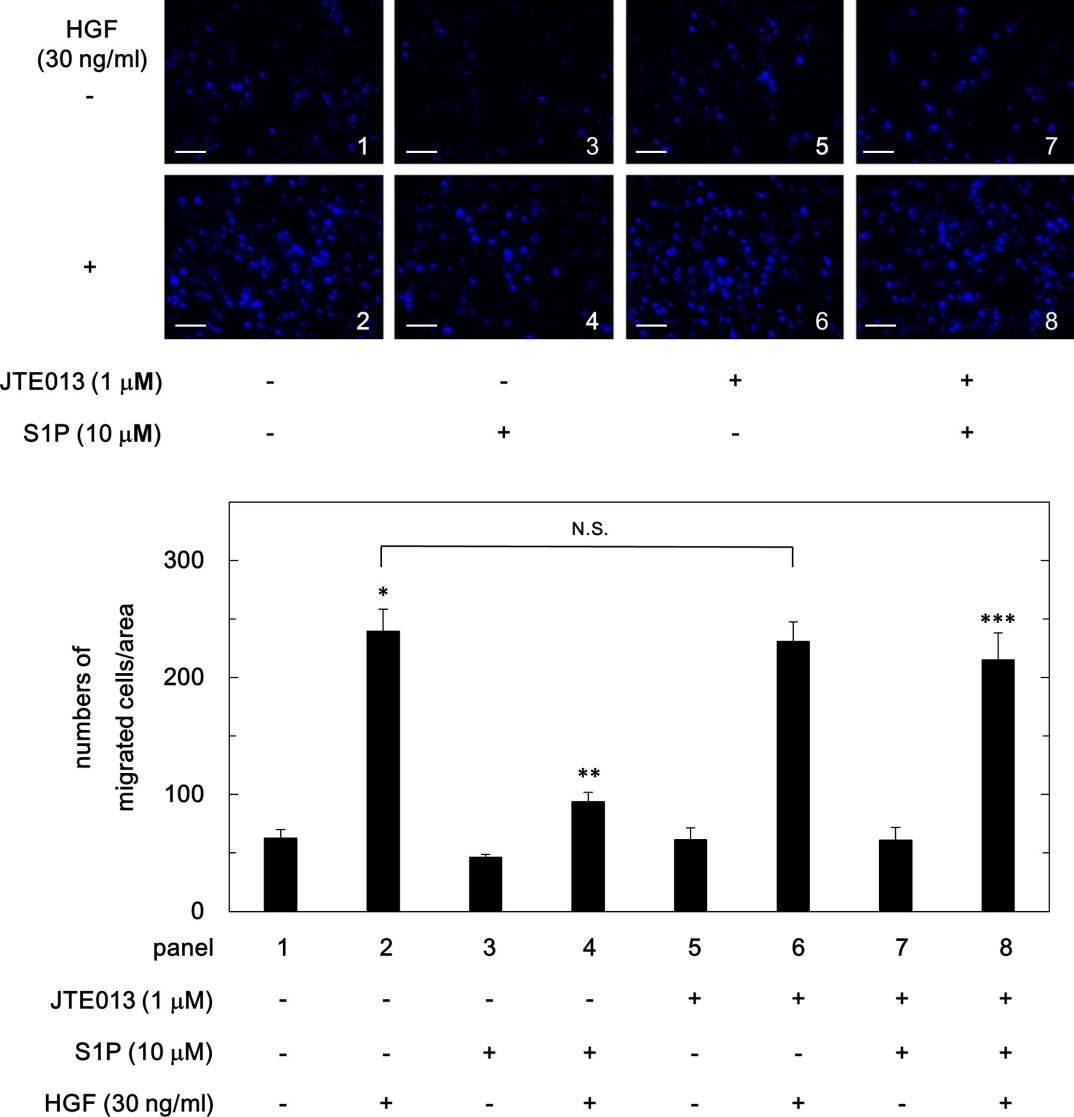
Effect of JTE013 on the suppression by S1P of the HGF-induced migration of HuH7 cells. The cells were pretreated with 1 μM of JTE013 for 10 min prior to 10 μM of S1P treatment for 60 min, and then stimulated by 30 ng/ml of HGF or vehicle for 23 h. The migrated cells were fixed with paraformaldehyde, and stained with DAPI for nucleus (blue signal). The cells were photographed by fluorescent microscopy at a magnification of 20× (upper panel) and counted (bar panel). Each value represents the mean ± SD of triplicate determinations from three independent cell preparations. *p<0.05 compared to the value of the control cells without HGF. **p<0.05 compared to the value of the cells with HGF alone. ***p<0.05 compared to the value of the cells with HGF and S1P pretreatment. N.S. designates no significant difference between the indicated pairs. Scale bar: 100 μm.

To further clarify whether or not the inhibitory effect of S1P on the HGF-induced migration of HuH7 cells is mediated through S1PR2, we examined the effect of JTE013, a selective antagonist of S1PR2 [32], on the suppression by S1P of the cell migration. As shown in Fig 6, JTE013, which did not affect the HGF-induced migration of HuH7 cells, markedly reversed the suppressive effect of S1P on the HGF-induced migration of HuH7 cells, almost to the level of HGF alone.

### Effect of S1PR2-siRNA on the suppression by S1P of HGF-induced migration of HuH7 cells

**Fig 7:**
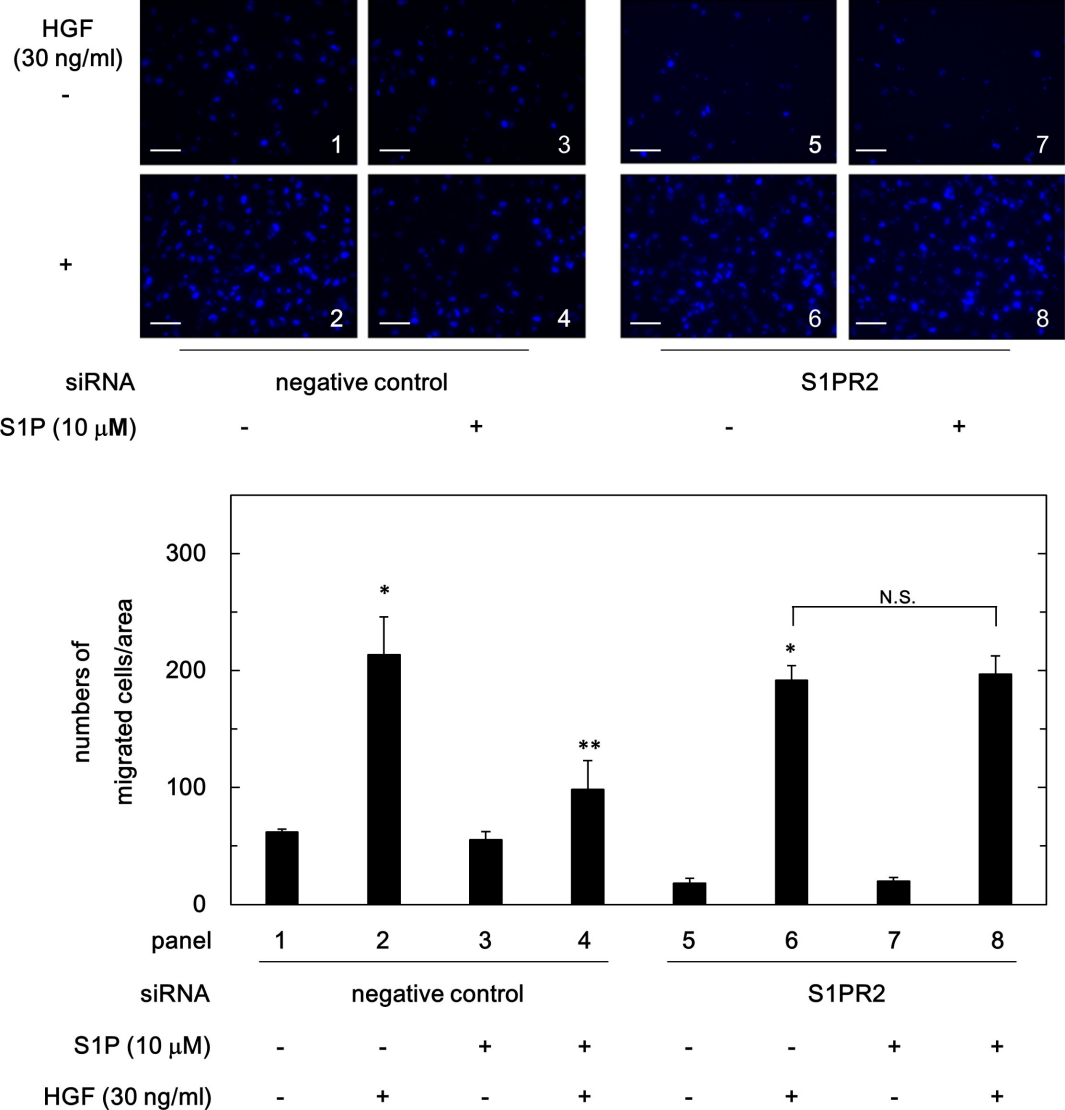
Effect of S1PR2-siRNA on the suppression by S1P of HGF-induced migration of HuH7 cells. The S1PR2-siRNA or negative control siRNA transfected HuH7 cells were pretreated with 10 μM of S1P for 60 min, and then stimulated by 30 ng/ml of HGF or vehicle for 23 h. The migrated cells were fixed with paraformaldehyde, and stained with DAPI for nucleus (blue signal). The cells were photographed by fluorescent microscopy at a magnification of 20× (upper panel) and counted (bar panel). Each value represents the mean ± SD of triplicate determinations from three independent cell preparations. *p<0.05 compared to the value of the control cells without HGF. **p<0.05 compared to the value of the cells with HGF alone. N.S. designates no significant difference between the indicated pairs. Scale bar: 100 μm.

To furthermore investigate the inhibitory effect of S1P on HGF-induced migration of HuH7 cells is mediated by S1PR2, we examined the effect of S1PR2-siRNA on the suppression by S1P of HGF-induced migration of HuH7 cells. As shown in Fig 7, S1P failed to suppress the HGF-induced migration of S1PR2-siRNA transfected HuH7 cells compared to that in the control cells.

## Discussion

It is recognized that HGF and its unique receptor, c-MET are involved in activation of the “invasive program” during HCC metastasis, and there is a correlation between c-MET overexpression and a higher incidence of intrahepatic metastasis of HCC [14]. Regarding intracellular signaling system in HCC, the MAPK pathway such as ERK, JNK and p38 MAPK, and the AKT pathway are known as frequently activated [1], and HGF/c-MET signal can be transduced through these pathways [14]. Especially, the AKT pathway is recognized as a predictor of HCC recurrence and a possible implication in the invasive phenotype [14]. Addition to the MAPK and AKT pathways, the Rho-kinase pathway reportedly plays an important role in the metastasis of HCC [21]. However, the detailed mechanism of how HGF induces HCC cell migration remains unclear. Therefore, we investigated the involvement of these signaling pathways in the HGF-induced migration of human HCC-derived HuH7 cells. We showed that p38 MAPK, JNK, AKT and Rho-kinase were involved in the HGF-induced migration of HuH7 cells. On the other hand, HGF-activated ERK did not affect the HuH7 cell migration. Although it is firmly established that ERK has an essential role in HCC progression [14,15], it seems unlikely that ERK plays a role in the migration of HCC cells. Based on our results, it is most likely that p38 MAPK, JNK, AKT and Rho-kinase, but not ERK play as positive regulators in the migration of HuH7 cells induced by HGF.

S1P, which is a product derived from cell membrane sphingolipids, extracellularly plays through specific receptors and regulates various biological cell functions including cell migration [7–9]. In the present study, we demonstrated that S1P significantly suppressed the HGF-induced migration of human HCC-derived HuH7 cells. Regarding S1P-effects on HCC cell migration, it has been shown that the level of sphingosine kinase 1, which catalyzes the phosphorylation of sphingosine to S1P, is increased in HCC tissues, and that sphingosine kinase 1 induces the migration of SMMC-7721 cells and HCCLM3 cells, the HCC cells, due to enhancement of S1P secretion [33]. In addition, S1P reportedly alone stimulates the migration of rat McA-RH7777 cells [34]. On the contrary, it has been reported that inhibition of expression of S1P lyase, which catalyzes S1P to an inactive product, leads to reduce migration and invasion of PLC/PRF/5 cells [35], and reduction of S1P levels in human HCC tissues is associated with early recurrence [35]. These reports are suggesting that S1P plays an inhibitory role in HCC cell migration consistent with our present result. It, therefore, seems likely that the discrepancies of the S1P-effects on HCC cell migration are due to the differences among cell types and species. Further investigations are necessary to elucidate the details behind the discordance in the effect of S1P on the migration of HCC cells.

**Fig 8:**
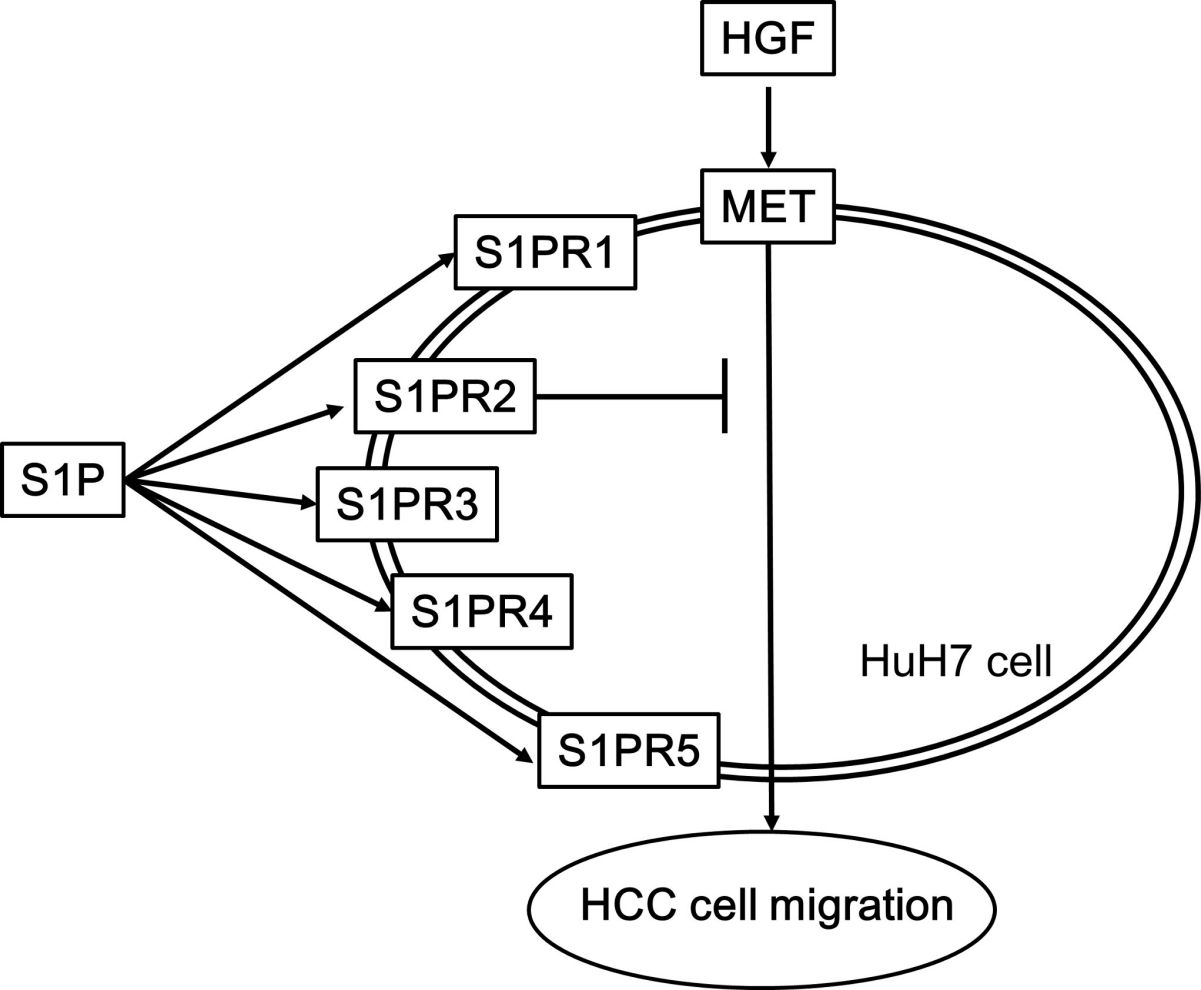
A schematic illustration of the regulatory mechanism of S1P in HGF-induced HCC cell migration. S1P, sphingosine 1-phosphate; HGF, hepatocyte growth factor; HCC, hepatocellular carcinoma, S1PR, sphingosine 1-phosphate receptor.

It is currently established that the extracellular effects of S1P are mediated through specific receptors of S1P, five known receptors (S1PR1-5) on cell membranes, and that S1PRs belongs to G-protein coupled receptors [7–9]. Since human liver tissue reportedly express all five receptors (S1PR1-5) [12], we confirmed that S1PR1-5 exist in human HCC-derived HuH7 cells. We next investigated which subtypes of S1PRs is involved in the suppression by S1P of the HGF-induced HuH7 cell migration. In this study, SEW2871 [27], CYM5541 [28], CYM50260 [29] and A981432 [30], a selective agonist of S1PR1, S1PR3, S1PR4 and S1PR5, respectively, failed to have a significant effect on the HuH7 cell migration. However, CYM5520, a selective agonist of S1PR2 [31], significantly reduced the HuH7 cell migration. These results suggest that only S1PR2 among S1PR1-5 mediates the S1P signaling in the migration of HuH7 cells. Furthermore, we demonstrated that JTE013, a selective antagonist of S1PR2 [32], remarkably reversed the reduction by S1P of HGF-induced HuH7 cell migration almost to the level of HGF stimulation alone. In addition, S1P hardly affected the HGF-induced migration of S1PR2-siRNA transfected HuH7 cells. Therefore, our results suggest that the suppressive effect of S1P on the cell migration is truly mediated through S1PR2 in HuH7 cells. Taking our findings into account as a whole, it is most likely that S1P reduces HGF-induced cell migration of HuH7 cells via S1PR2. Our findings may provide a novel potential of S1PR2 to therapeutic strategy against metastasis of HCC. The potential mechanism for regulation of HCC migration by S1P shown here is summarized in Fig 8. Further investigations are required to clarify the exact mechanism of S1P and responsive S1PR in HCC cell migration.

In conclusion, our findings strongly suggest that S1PR2-mediated S1P signal suppresses HGF-induced HCC cell migration.

## Supporting information

S1 Fig Dose dependent effect of HGF on the migration of HuH-7 cells. The cells were stimulated by various doses of HGF for 23 h. The migrated cells were fixed with paraformaldehyde, and stained with DAPI for nucleus (blue signal). The cells were photographed by fluorescent microscopy at a magnification of 20× (upper panel) and counted (bar panel). Each value represents the mean ± SD of triplicate determinations from three independent cell preparations. *p<0.05 compared to the value of the control cells without HGF. Scale bar: 100 μm.
(TIF)
