# Supplementary figures and images for: Sphingosine 1-phosphate (S1P) reduces hepatocyte growth factor-induced migration of hepatocellular carcinoma cells via S1P receptor 2
Source: PLoS One. 2018 Dec 13;13(12):e0209050. doi: 10.1371/journal.pone.0209050 (PMC6292590; doi:10.1371/journal.pone.0209050)

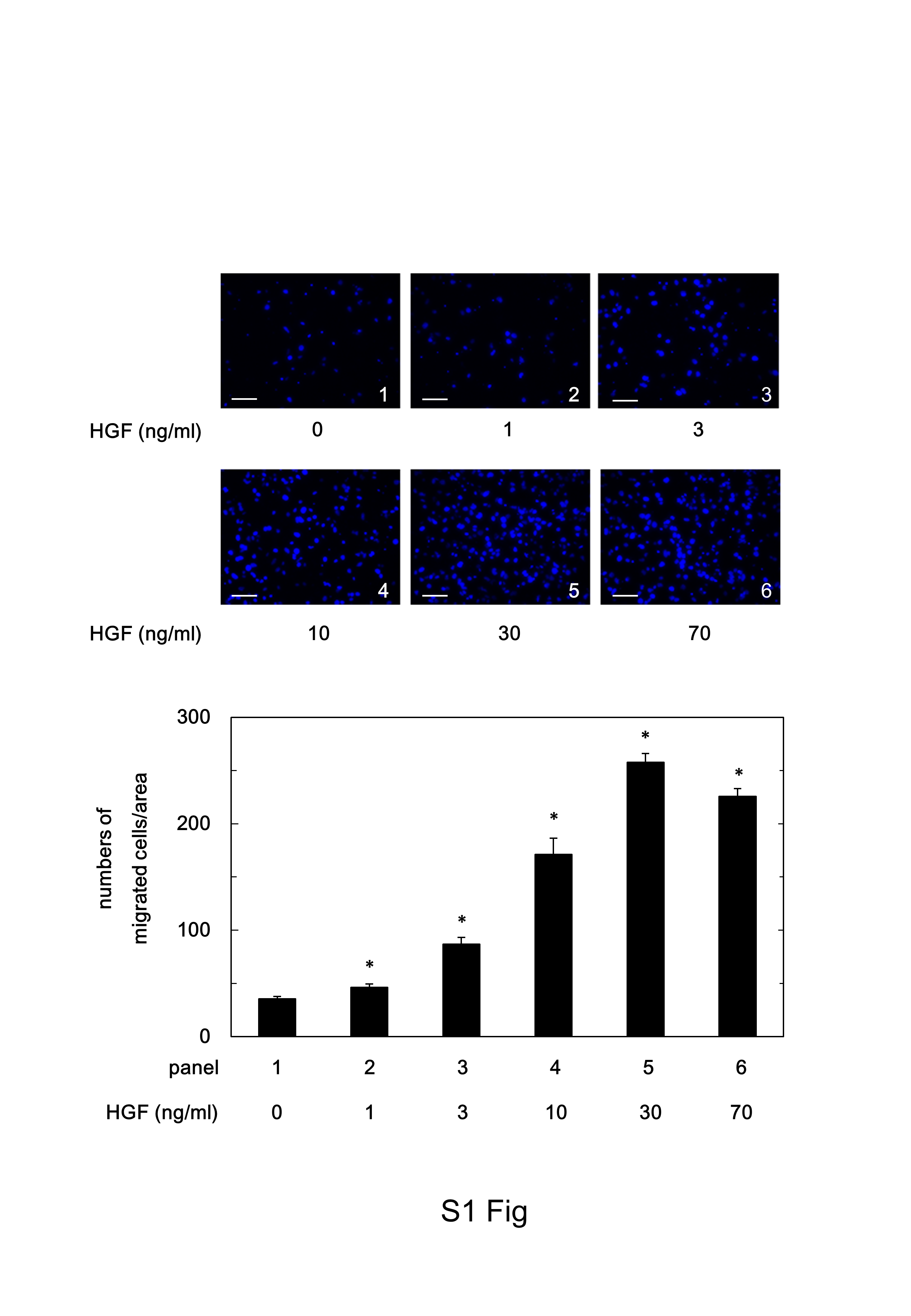

Supplement: S1 Fig — The cells were stimulated by various doses of HGF for 23 h. The migrated cells were fixed with paraformaldehyde, and stained with DAPI for nucleus (blue signal). The cells were photographed by fluorescent microscopy at a magnification of 20× (upper panel) and counted (bar panel). Each value represents the mean ± SD of triplicate determinations from three independent cell preparations. *p<0.05 compared to the value of the control cells without HGF. Scale bar: 100 μm. (TIF) [file pone.0209050.s001.tif]
